# Depression and the Risk of Myocardial Infarction and Coronary Death: A Meta-Analysis of Prospective Cohort Studies

**DOI:** 10.1097/MD.0000000000002815

**Published:** 2016-02-12

**Authors:** Qing Wu, Juliana M. Kling

**Affiliations:** From the Nevada Institute of Personalized Medicine (QW), and Department of Environmental and Occupational Health, School of Community Health Sciences (QW), University of Nevada Las Vegas, Las Vegas, NV, and Division of Women's Health Internal Medicine (JMK), Mayo Clinic, Scottsdale, AZ.

## Abstract

Findings regarding the association between depression and risk of coronary heart disease are inconsistent. We aimed to assess the association between depression and risk of myocardial infarction (MI) and coronary death through a meta-analysis.

We performed an electronic literature search of MEDLINE, EMBASE, PsycINFO, ISI Web of Science, and Scopus databases through August 1, 2015, and manual search of the references of the eligible papers and related review articles. Two investigators independently conducted study selection and data abstraction. Disagreement was resolved by consensus. Confounder-adjusted hazard ratios (HRs) were pooled using a random-effects model. Heterogeneity was evaluated using the Cochran Q statistic and Higgins index. Publication bias was assessed by funnel plot and Egger test. Study quality was appraised with the Newcastle-Ottawa Scale.

Among 19 eligible cohort studies including 323,709 participants, 8447 cases of MI and coronary death were reported during follow-up ranging from 4 to 37 years. The pooled adjusted HRs for patients with depression (vs those without) were 1.22 (95% CI, 1.13–1.32) for combined MI and coronary death, 1.31 (95% CI, 1.09–1.57) for MI alone (9 studies), and 1.36 (95% CI, 1.14–1.63) for coronary death alone (8 studies). The increased risk of MI and coronary death associated with depression was consistent using modified inclusion criteria, across most subgroups, and after adjusting for possible publication bias.

Depression is associated with a significantly increased risk of MI and coronary death. Effective prevention and treatment of depression may decrease such risk.

## Abstract 2

Supplemental Digital Content is available in the text

## INTRODUCTION

Coronary heart disease (CHD) remains the leading cause of death in the United States, United Kingdom, and other Western countries, although its mortality rates have decreased slightly because of advances in health care. In 2010, an estimated 379,559 deaths in the United States were caused by CHD, ≈1 in every 6 deaths.^1^ In the United Kingdom, this number is more than 65,000, which is more than deaths from any other disease.^2^ In many developing countries, mortality and morbidity from CHD have increased exponentially. In 2008, an estimated 7.3 million global deaths resulted from CHD;^3^ thus, it is becoming the leading cause of death worldwide.

Another common disorder, depression, affects 26% of women and 18% of men in the United States.^4^ Many studies have examined the effects of depression on the risk of CHD, especially as a potential modifier of myocardial infarction (MI) and coronary death. Results from previous meta-analyses and reviews, however, have been inconsistent.^5–10^ These meta-analyses included either studies with other than a prospective design or a different subset of available studies, or studies with heterogeneous outcomes of heart diseases. None have offered a comprehensive review of all the relevant evidence in prospective cohort studies to investigate the association between depression and the risk of MI or death due to CHD.

The aims of this meta-analysis were to quantitatively assess all qualified prospective cohort studies that have examined the effect of depression on the risk of MI or death due to coronary diseases and to gather more accurate and precise information about this effect.

## METHODS

This meta-analysis followed the MOOSE guidelines,^11^ with reference to the PRISMA statement,^12^ regarding the literature search, inclusion criteria, study selection, data abstraction, study appraisal, and data analysis. Articles in other languages were reviewed and evaluated by multilingual investigators; the same criteria and assessment methods were employed. Institutional review board approval was not required because this meta-analysis only used published data and no patient consent was needed. We preregistered our protocol on PROSPERO (CRD42015026892) and is available at: http://www.crd.york.ac.uk/PROSPERO/display_record.asp?ID=CRD42015026892.

### Eligibility Criteria

Because of the bidirectional relationship between depression and CHD, this meta-analysis only included original prospective cohort studies that evaluated the effects of depression on the risk of MI or CHD death, in which depression is the predictor and MI and CHD death is the outcome. Case–control and cross-sectional studies were excluded.

Eligible exposures were unipolar depression assessed by clinical diagnosis or depressive mood measured by a standardized psychometric tool. In this meta-analysis, the term “depression” refers to clinical depression, depressive disorder, and depressive mood. Bipolar depression and bipolar depressive disorder were excluded.^13^ Eligible outcomes were fatal or nonfatal MI or death due to CHD. We excluded angina pectoris because some studies have demonstrated that some patients with depression report chest pain but have normal coronary arteries.^14,15^

Included studies were required to have a control group (no depression) and to report adjusted hazard ratio (HR) or relative risk (RR) of outcomes between depressed and nondepressed participants. Studies that analyzed depression as a continuous variable and did not report HR or RR between a depressed and nondepressed group were excluded.

### Literature Search

A literature search of MEDLINE was conducted, using both Ovid and PubMed from January 1966 through August 2015, without language restrictions. Two major search themes were combined using the “and” Boolean operator. The first theme, depression, combined the exploded versions of the Medical Subject Headings “depression” and “depressive disorder.” The second theme, MI and coronary death, combined exploded versions of the Medical Subject Heading terms “coronary disease” and “myocardial infarction.” The key word “coronary heart disease(s)” was also used in the search. The same search strategy was used with the databases EMBASE (from 1988), PsycINFO (from 1806), ISI Web of Science (from 1975), and Scopus (from 1960), with the last search performed on August 1, 2015. Ovid AutoAlert automatically updated the MEDLINE literature search to November 3, 2015. We then manually searched the reference lists of eligible papers and related review articles retrieved in the electronic search. We searched abstracts listed in WorldCat Dissertations and Theses using the key words “depressive disorder,” “depression,” and “heart disease(s).” An experienced medical librarian was consulted for the literature search. We also used Google to search for conference materials, abstracts, and unpublished data. The 2 investigators independently examined reference lists manually from included original studies^16–34^ and review articles.^5–9,35,36^

### Study Selection

All references from electronic database searches, including abstracts, were merged and stored in Endnote (Thomson Reuters), Philadelphia, PA, USA; duplicates were removed by experienced librarians. The 2 investigators independently screened the title and abstract of each reference in the EndNote database to identify potentially eligible studies and then independently reviewed the full contents to determine their eligibility for this meta-analysis. Agreement between investigators was assessed with the κ statistic. Disagreement regarding eligibility was resolved by consensus.

### Study Appraisal

We used the Newcastle-Ottawa Scale^37^ to assess the methodological quality of included studies (eTable 1). These items were prespecified in the Research Electronic Data Capture (REDCap) tool hosted at Mayo Clinic.^38^ REDCap is a secure, web-based application designed to support data capture for research studies. As recommended by the MOOSE study group,^11^ the quality scores were not used as weights in the analyses but as differentiators in the subgroup analysis (score <7 vs ≥7).

### Data Abstraction

All data were independently abstracted by the 2 investigators using the REDCap tool.^38^ Disagreement or uncertainty was resolved by discussion. The following information was recorded: authors, title, year published, and study country; study design, sample size, and sampling methods; distribution by age, sex, and race; exposures and their measurement methods; outcomes and their validation methods; duration of follow-up; adjusted risk factors; and HR or RR of MI or CHD death associated with depression. If the original study gave multiple estimates of the same outcome from the nested regression models, we used the estimate adjusting for the most confounders. We did not contact authors because no further information was needed for eligible studies.

### Statistical Analysis

HR was used as a measure of the association between depression and the risk of MI or CHD death. RR was considered equivalent to HR. The confounder-adjusted HR or RR was the primary outcome. The HRs or RRs of individual studies were transformed to natural logarithms to stabilize the variance and normalize the distributions.^39^ For the overall pooled analysis, the longest duration of follow-up was used in cases of multiple data points over time. To pool overall effect size, each study was weighted by the reciprocal of its variance. Outcome heterogeneity across studies was assessed with the Cochran Q statistic (*P* < 0.1 indicated statistically significant heterogeneity)^40^ and Higgins index (*I*^2^) (values of 75%, 50%, and 25% indicated high, medium, and low heterogeneity, respectively).^41^ Due to heterogeneity among studies, all pooled analyses used random-effects models.

Multiple sensitivity analyses were conducted to evaluate the findings of the meta-analysis using different assumptions. We conducted several subgroup analyses based on the quality of study methods, sex, mean age of participants at baseline, sample size, duration of follow-up and others. A cumulative meta-analysis was conducted by undertaking sequential random-effects pooling, starting with the earliest, eligible study. Each successive meta-analysis then summarized all included studies in the preceding years. Results were presented chronologically in a forest plot to demonstrate the impact of adding studies on the pooled estimate. No multivariate meta-regression analysis was performed because no significant difference was found between groups in the subgroup analysis.

To examine for possible publication bias, we used funnel plots and the Egger test.^42^ Furthermore, the Duval and Tweedie nonparametric trim-and-fill method^43^ was used to assess and adjust for the potential effects of unpublished studies on the pooled estimate.

The absolute risk differences associated with depression were calculated by multiplying the background incidence rate of MI and CHD death in the general US population by estimated HR – 1. We calculated population-attributable risk as: *P*_e_(HR_e_ – 1)/[1 + *P*_e_(HR_e_ – 1)]; where *P*_e_ is the prevalence of depression and HR_e_ was calculated from the present meta-analysis. All data analyses were performed with Stata 10.0 software (STATA Corp, College Station, TX, USA).

## RESULTS

### Literature Search and Study Selection

**FIGURE 1:**
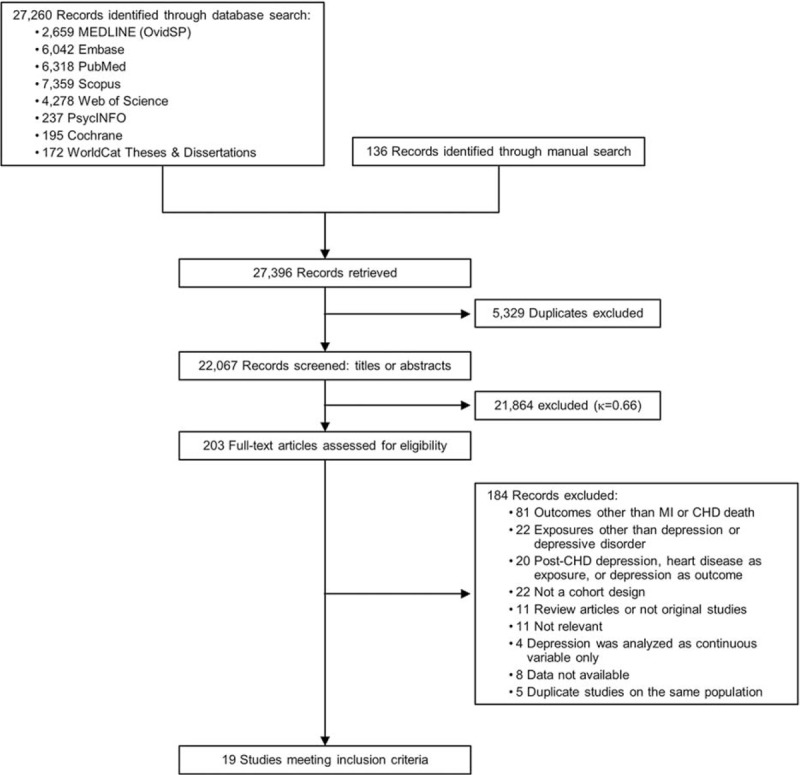
Study selection flow diagram. Selection of cohort studies for meta-analysis of association between depression and risk of myocardial infarction (MI) or death due to coronary heart disease (CHD).

The study flow diagram is shown in Figure 1. After eliminating duplicate publications, we identified 22,067 potential articles. Initial screening of titles and abstracts resulted in 203 full-text articles assessed for eligibility, with modest agreement between the 2 investigators (κ = 0.66). After full review of these articles, 19 studies (all published in English) met the inclusion criteria,^16–34^ with good agreement between the investigators (κ = 0.92).

### Study Characteristics

The characteristics of the study participants and the designs of the cohort are summarized in Table 1 and eTable 2. Among the cohort studies, 11 were conducted in the United States, 8 included only women, and 8 included only patients aged 65 years and older. The number of participants ranged from 1190 (18) to 63,469 (28), and the number of cases of MI and coronary death ranged from 20 (19) to 2111 (33). The mean follow-up period ranged from 4 (22) to 37 (29) years.

**TABLE 1:**
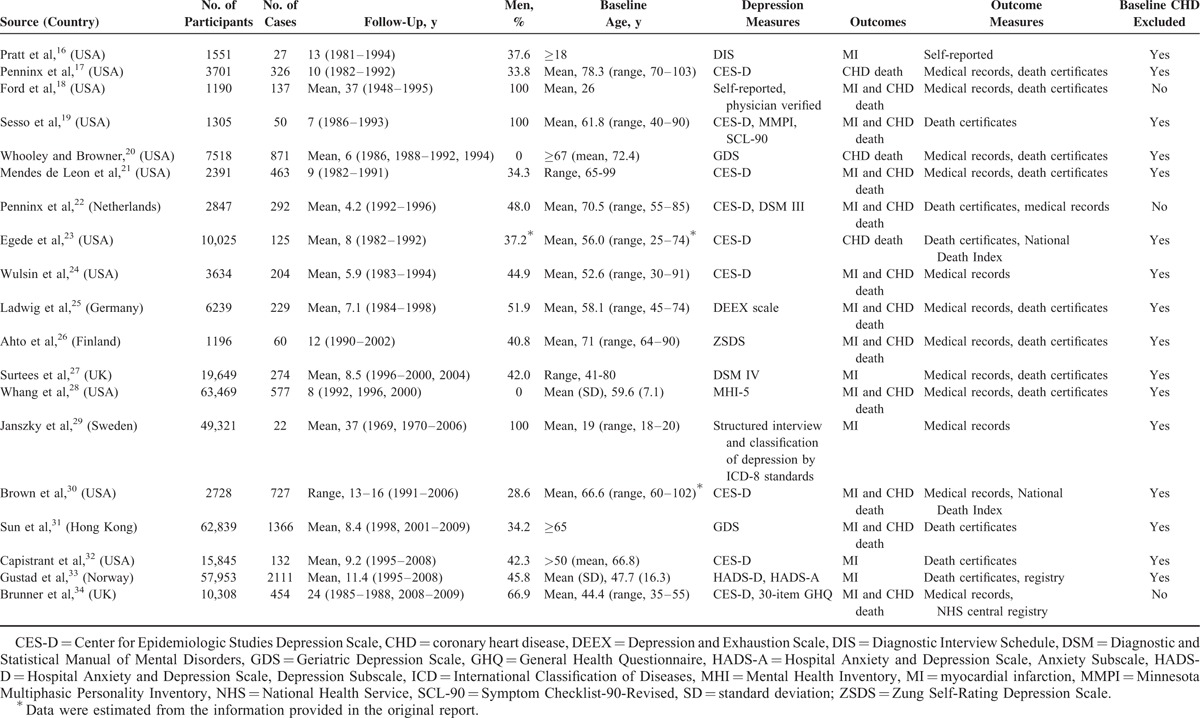
Characteristics of Studies Included in the Meta-Analysis

### Overall Analysis

**FIGURE 2:**
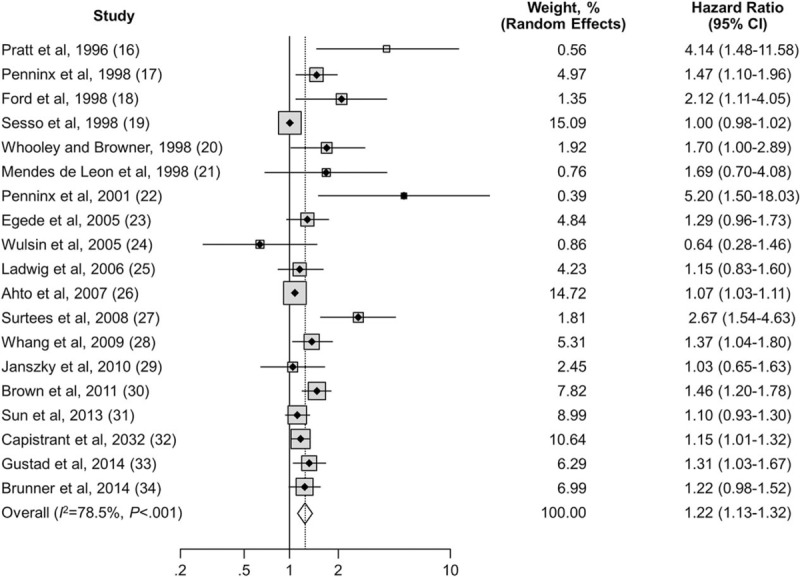
Effect of depression on the risk of MI and CHD death combined. Hazard ratios (95% CIs) are shown; sizes of data markers are proportional to the weight of every study in the forest plot. CHD = coronary heart disease, MI = myocardial infarction.

Data were analyzed for a total of 323,709 persons, a majority of whom were women (58.1%); 8447 cases of MI and CHD death were reported during follow-up. The Cochran Q statistic (*P* < 0.001) and the Higgins *I*^2^ index (78.5%) indicated that there was heterogeneity among the 19 studies. Figure 2 shows the HR of MI and death due to CHD associated with depression in each study and overall. Compared with nondepressed persons, depressed people had an overall HR of 1.22 (95% CI, 1.13–1.32; *P* < 0.001). Of the 19 eligible studies, 9 used MI as an outcome and 8 used coronary death as an outcome. Overall, depression was associated with a 31% increase in the risk of MI (HR, 1.31; 95% CI, 1.09–1.57; *P* < 0.001) (eFigure 1); depression was associated with a 36% increase in the risk of coronary death (RR, 1.36; 95% CI, 1.14–1.63; *P* < 0.001) (eFigure 2).

On the basis of 2015 heart disease statistics,^44^ the absolute risk difference associated with depression in the United States was estimated to be 51 cases of MI and 40 coronary deaths per 100,000 persons per year. According to the most recent data from the National Institute of Mental Health, an estimated 16 million adults in the United States (6.9%) meet the definition for current depression.^45^ Using the risk estimates from the present meta-analysis, we estimate that 2.4% of coronary deaths (n = 9000) and 2.1% of MI cases (n = 13,300) in the United States could be attributable to depression.

### Sensitivity Analysis and Subgroup Analysis

Table 2 summarizes results of sensitivity analysis; the pooled estimated HR changed little when studies with different inclusion criteria were excluded. The cumulative meta-analysis demonstrated that the evidence was consistent since 1998 (eFigure 3). The pooled HR estimates and their CIs stabilized from 2013 and remained unchanged, even after adding 2 large studies.^33,34^ This result suggests that the addition of a future study, even if it included thousands of participants, would add little to the cumulative body of evidence. Table 3 summarizes the pooled estimates of HR associated with depression in subgroups of studies according to mean age at baseline, sex, study methodology quality, sample size, controlling key confounders, publication year, and geographic location. Depression significantly increased the risk of MI and CHD death in all subgroups except in women (*P* = 0.08) and studies that had mean follow up less than eight years (*P* = 0.27). The increased risk was more evident in several subgroups (Table 3), but no significant between-group differences were observed. Moderate to high heterogeneities were observed in most of these analyses.

**TABLE 2:**
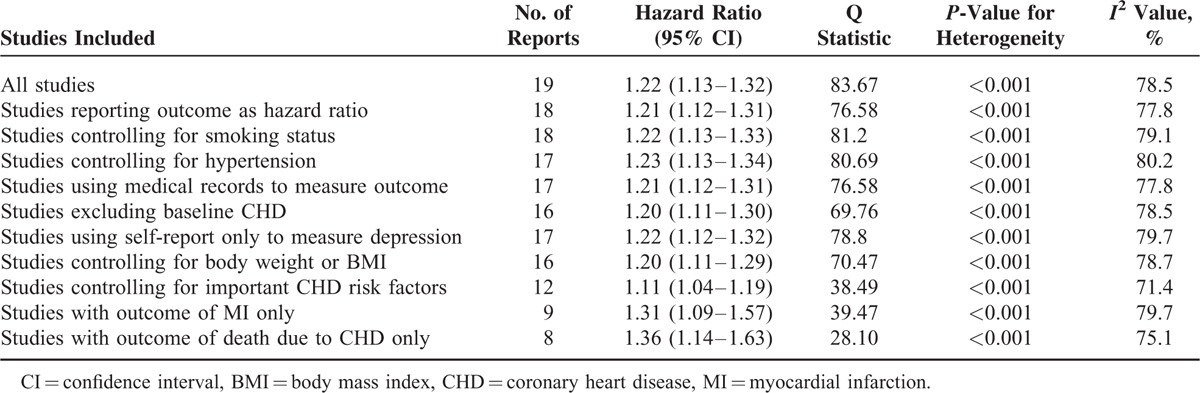
Sensitivity Analyses for Different Inclusion Criteria

**TABLE 3:**
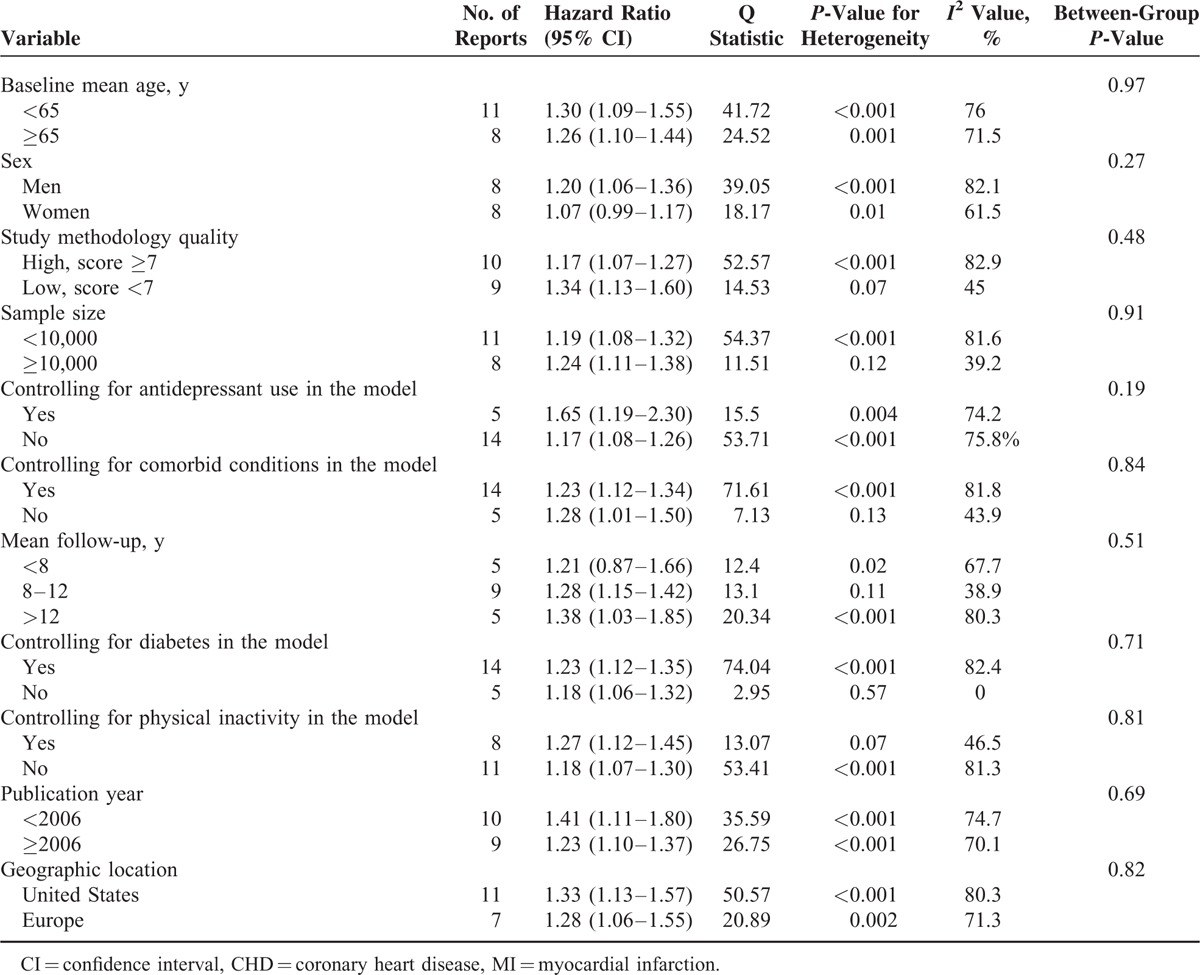
Stratified Analyses of Hazard Ratio for MI and CHD Death Associated With Depression by Subgroup

### Analysis of Publication Bias

**FIGURE 3:**
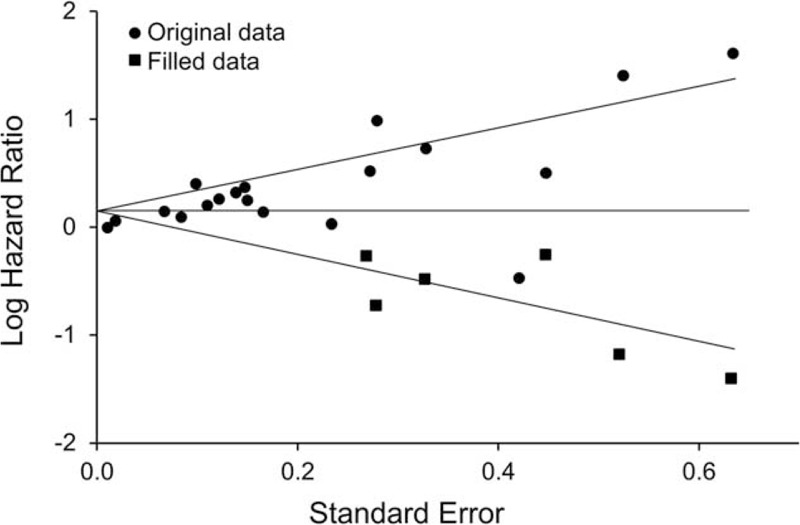
Funnel plots for detection of publication bias.

Publication bias was suspected in this meta-analysis, as indicated by the funnel plot (Figure 3), which revealed asymmetry. The Egger test was significant (t = 5.95; *P* < 0.01). We conducted a sensitivity analysis by using the trim-and-fill method; a symmetrical funnel plot was produced with 6 imputed studies. The overall estimate was smaller after trim-and-fill correction, but it remained statistically significant (HR, 1.17; 95% CI, 1.08–1.27; *P* < 0.001).

## DISCUSSION

Our meta-analysis showed that depression is prospectively associated with a significant increase in the risk of MI and coronary death. Furthermore, the increased risk associated with depression persisted and remained statistically significant in sensitivity analyses on the basis of various inclusion criteria and in all subgroup analyses stratified by various participant and study characteristics. In addition, the cumulative meta-analysis also demonstrated that our results are robust and consistent. A positive association was found when pooled analysis was performed for MI and coronary death separately. Although the risk associated with depression found in this meta-analysis was moderate, because of the high prevalence of depression, the estimated absolute risk differences associated with depression were substantial, with 13,300 cases for MI and 9000 cases for coronary death in the United States alone. Given that depression is prevalent worldwide, the findings of our meta-analysis have important implications for global public health.

The results of our meta-analysis are consistent with those of 2 previous meta-analyses that assessed the confounder-adjusted effect of depression on fatal CHD and incident MI. The first, by Rugulies,^5^ included 11 studies published before 1999, and the overall RR was 1.64 (95% CI, 1.29–2.08). The second, by Nicholson et al,^8^ presented an overall confounder-adjusted effect from 12 studies published before 2004, the estimate of overall RR was 1.90 (95% CI, 1.49–2.42). Although both meta-analyses presented larger overall estimates than our study, their results were restricted because these meta-analyses included crude RR or age-adjusted RR, and these unadjusted confounders tend to exaggerate the estimate. Due to incomplete and biased availability of adjustments for coronary risk factors, Nicholson et al suggested additional evaluation is warranted to establish the association between depression and CHD risk. Our meta-analysis attempted to minimize this problem by only including studies which adjusted for most of the important confounders. In addition, both previous meta-analyses had a substantially smaller sample size than our study. Our meta-analysis, with 7 times more cases, provides strong evidence that depression is associated with an increased risk of MI and coronary death. Finally our cumulative meta-analysis also demonstrated that our pooled estimate stabilized in 2013 and remained unchanged after 2 new studies were added,^18,19^ which suggests that even adding a future large study would have little impact on our estimate. Other earlier meta-analyses and reviews focused on different outcomes, either incidence of other CHD events^6,7^ or broad cardiovascular disease outcomes.^9^ A more recent meta-analysis by Gan et al,^10^ which combined studies with 2 different outcomes—development of various CHDs and death due to CHD—was restricted because it had omitted several major eligible studies^21,23,25,27^ but included duplicate reports from the same NHANES I study population^46,47^ and included ineligible studies with RR from continuous depression scores.^48,49^ By contrast, our meta-analysis focused on MI and coronary death only, excluded angina pectoris and other nondefinitive CHD outcomes, and contained all qualified studies, including those omitted by previous meta-analyses. Therefore, to our knowledge, the present meta-analysis includes all eligible data in assessing the effect of depression on risk of MI and coronary death.

The underlying mechanism of how depression contributes to CHD has not been fully elucidated. First, increasing evidence suggests that the association between depression and CHD prognosis may be explained by several biological mechanisms including inflammation, platelet reactivity, autonomic dysregulation, sleep architecture disruption, circadian rhythm disruption, anabolic/catabolic hormone imbalance, and others.^36^ A meta-analysis by Howren et al^50^ found that depression is associated with increased values of C-reactive protein, interleukin-1, and interleukin-6 and that these inflammatory factors were found to be linked to an increased risk of CHD and CHD mortality.^51^ Second, many poor health behaviors are associated with depression, such as smoking,^52^ increased alcohol consumption,^53^ and physical inactivity.^54^ These poor health behaviors are also well-established risk factors for CHD. Third, depression is correlated with obesity, diabetes mellitus, hypertension, and other major comorbid conditions, which themselves are also associated with increased risk of CHD. Controlling for these comorbid conditions in the current meta-analysis somewhat attenuated the association between depression and MI/coronary death, which indicates that comorbid conditions may confound the association between depression and CHD. Finally, use of antidepressants may contribute to the association we observed. Although the association was not attenuated by adjusting for antidepressant use, we still could not rule out this possibility because antidepressant use usually indicates more severe depression; however, information on type, duration, and dose of antidepressant medication use was not available in the original reports for further investigation.

This study has several limitations. First, a multivariate meta-regression analysis was not performed, because no significant difference was found between groups in the subgroup analyses. However, various subgroup analyses were conducted to investigate variations in the association among many subgroups of interest. Second, publication bias is suspected in this meta-analysis, as indicated by funnel plot and Egger test, but we adjusted for this using trim-and-fill method, after which the pooled HR remained significant (although the association was slightly attenuated). Furthermore, most original studies used self-report scales to measure depression, which may cause misclassification bias and lead to underestimation of the risk associated with MI and coronary death in this meta-analysis. Finally, some studies included in our meta-analysis lacked information on medication use. The role of medications in the association between depression and risk of MI and CHD death needs further investigation.

In conclusion, this meta-analysis of prospective cohort studies suggests that depression is significantly associated with an increased risk of MI and coronary death. Because of the high prevalence of depression and the incidence of MI and CHD mortality worldwide, the observed association between depression and MI and coronary death has important implications for public health. Prevention and treatment of depression may substantially decrease the risk of MI and coronary death globally. Further studies are warranted to investigate the underlying mechanisms for how depression causes increased risk of MI and CHD death.

## Supplementary Material

Supplemental Digital Content
