# Supplementary material for: Depression and the Risk of Myocardial Infarction and Coronary Death: A Meta-Analysis of Prospective Cohort Studies
Source: Medicine (Baltimore). 2016 Feb 12;95(6):e2815. doi: 10.1097/MD.0000000000002815 (PMC4753948; doi:10.1097/MD.0000000000002815)
Supplement: Supplemental Digital Content [file medi-95-e2815-s001.doc]

**eTable 1. Quality of Included Studies**

| **Reference** | **Representativeness of the exposed cohort** | **Selection of the nonexposed cohort** | **Ascertainment of exposure** | **Demonstration that outcome of interest was not present at start of study** | **Comparability of cohorts on the basis of the design or analysis** | **Assessment of outcome** | **Was follow-up long enough for outcomes to occur** | **Adequacy of follow-up of cohorts** | **Total score** |
| --- | --- | --- | --- | --- | --- | --- | --- | --- | --- |
| Pratt et al, 1996 (1) | 1 | 1 | 1 | 1 | 2 | 0 | 1 | 0 | 7 |
| Penninx et al, 1998 (2) | 1 | 1 | 1 | 1 | 2 | 1 | 1 | 0 | 8 |
| Ford et al, 1998 (3) | 0 | 1 | 0 | 1 | 1 | 1 | 1 | 0 | 5 |
| Sesso et al, 1998 (4) | 0 | 1 | 1 | 1 | 2 | 1 | 1 | 0 | 7 |
| Whooley and Browner, 1998 (5) | 0 | 1 | 0 | 1 | 1 | 1 | 1 | 1 | 6 |
| Mendes de Leon et al, 1998 (6) | 1 | 1 | 1 | 1 | 2 | 1 | 1 | 0 | 8 |
| Penninx et al, 2001 (7) | 1 | 1 | 0 | 1 | 2 | 1 | 1 | 0 | 7 |
| Egede et al, 2005 (8) | 1 | 1 | 0 | 1 | 1 | 1 | 1 | 0 | 6 |
| Wulsin et al, 2005 (9) | 1 | 1 | 0 | 1 | 1 | 1 | 1 | 0 | 6 |
| Ladwig et al, 2006 (10) | 1 | 1 | 0 | 1 | 1 | 1 | 1 | 0 | 6 |
| Ahto et al, 2007 (11) | 1 | 1 | 1 | 1 | 1 | 1 | 1 | 0 | 7 |
| Surtees et al, 2008 (12) | 1 | 1 | 0 | 1 | 1 | 1 | 1 | 0 | 6 |
| Whang et al, 2009 (13) | 1 | 1 | 0 | 1 | 1 | 1 | 1 | 0 | 6 |
| Janszky et al, 2010 (14) | 0 | 1 | 1 | 1 | 1 | 1 | 1 | 0 | 6 |
| Brown et al, 2011 (15) | 1 | 1 | 0 | 1 | 2 | 1 | 1 | 0 | 7 |
| Sun et al, 2013 (16) | 1 | 1 | 1 | 1 | 1 | 1 | 1 | 1 | 8 |
| Capistrant et al, 2013 (17) | 1 | 1 | 0 | 1 | 1 | 1 | 1 | 1 | 7 |
| Gustad et al, 2014 (18) | 1 | 1 | 0 | 1 | 1 | 1 | 1 | 1 | 7 |
| Brunner et al, 2014 (19) | 1 | 1 | 0 | 0 | 1 | 1 | 1 | 1 | 6 |

**eTable 2. Other Characteristics of Included Studies**

|  |  |  |  | **Outcome Reported** | | |
| --- | --- | --- | --- | --- | --- | --- |
| **Reference** | **Statistical Model** | **Risk Expression** | **Adjusted Covariates** | **Overall**^a^ | **MI** | **Death Due to CHD** |
| Pratt et al, 1996 (1) | Logistic regression | OR | Heart disease, hypertension, diabetes, use of tobacco, alcohol, other substances, education, household income, marital status | Impute from MI | Y | N |
| Penninx et al, 1998 (2) | Cox proportional hazards regression | HR | Age, sex, smoking status, alcohol intake, BMI, blood pressure/hypertension, hyperlipidemia, diabetes mellitus, stroke, cancer, physical disability | Y | N | N |
| Ford et al, 1998 (3) | Cox proportional hazards regression | HR | Changes in smoking status, coffee drinking, alcohol intake, hyperlipidemia, clinical depression, hypertension, and diabetes were included as time-dependent variables | Impute from MI | Y | N |
| Sesso et al, 1998 (4) | Cox proportional hazards regression | HR | Age, blood pressure, cholesterol, smoking status, weight, BMI, diabetes, alcohol intake, family history of CHD | Impute from MI | Y | N |
| Whooley and Browner, 1998 (5) | Cox proportional hazards regression | HR | Age, marital status, education, medical history (stroke, MI, COPD), perceived health status, smoking, alcohol, benzodiazepine usage, barbiturate usage, thiazide diuretic usage, estrogen usage, hypertension, BMI, physical activity, cognitive function | Impute from CHD death | N | Y |
| Mendes de Leon et al, 1998 (6) | Cox proportional hazards regression | HR | Smoking status, diabetes, angina, blood pressure, physical functioning, sex, education, ethnicity, marital status, income | Y | Y | Y |
| Penninx et al, 2001 (7) | Cox proportional hazards regression | HR | Age, sex, education level, smoking, alcohol intake, BMI, blood pressure, diabetes, stroke, lung disease, cancer | Impute from CHD death | N | Y |
| Egede et al, 2005 (8) | Cox proportional hazards regression | HR | Age, sex, race/ethnicity, poverty: income ratio, education, marital status, smoking, physical activity, BMI, aspirin use, and comorbid medical conditions at baseline (cancer, hypertension, heart disease, and stroke) | Impute from CHD death | N | Y |

**eTable 2 (continued)**

|  |  |  |  | **Outcome Reported** | | |
| --- | --- | --- | --- | --- | --- | --- |
| **Reference** | **Statistical Model** | **Risk Expression** | **Adjusted Covariates** | **Overall** | **MI** | **Death Due to CHD** |
| Wulsin et al, 2005 (9) | Cox proportional hazards regression | HR | Age, hypertension, diabetes, cigarette smoking status, alcohol use, serum total cholesterol, BMI | Y | N | N |
| Ladwig et al, 2006 (10) | Cox proportional hazards regression | HR | BMI, cholesterol, smoking status, education level, social support, marital status, happiness, sleep problems | Y | N | N |
| Ahto et al, 2007 (11) | Cox proportional hazards regression | HR | Prior CHD, age, marital status, social status, level of education, previous occupation, functional abilities, smoking, hypertension, diabetes, BMI | Impute from CHD death | N | Y |
| Surtees et al, 2008 (12) | Cox proportional hazards regression | HR | Age, sex, smoking, systolic blood pressure, cholesterol, physical activity, BMI, diabetes, social class, heavy alcohol use, antidepressant medication | Impute from CHD death | N | Y |
| Whang et al, 2009 (13) | Cox proportional hazards regression | HR | Age, beginning year of follow-up, smoking status, BMI, alcohol, menopausal status and postmenopausal hormone use, use of aspirin, multivitamin use, vitamin E supplement use, hypercholesterolemia, family history of MI, history of stroke, n-3-fatty acid intake, alphalinolenic acid intake, physical activity, nonfatal CHD during follow-up, hypertension and diabetes | Y | Y | Y |

**eTable 2 (continued)**

|  |  |  |  | **Outcome Reported** | | |
| --- | --- | --- | --- | --- | --- | --- |
| **Reference** | **Statistical Model** | **Risk Expression** | **Adjusted Covariates** | **Overall** | **MI** | **Death Due to CHD** |
| Janszky et al, 2010 (14) | Cox proportional hazards regression | HR | Anthropometrics, diabetes, blood pressure, smoking, alcohol use, physical activity, socioeconomic position, family history, geographic area, BMI | Impute from MI | Y | N |
| Brown et al, 2011 (15) | Cox proportional hazards regression | HR | Age, sex, race, history of smoking, cholesterol level, body weight, history of diabetes or hypertension, and chronic conditions: diabetes, hypertension, atherosclerotic vascular disease, CHD, congestive heart failure, and cerebrovascular disease | Y | Y | Y |
| Sun et al, 2013 (16) | Cox proportional hazards regression | HR | Age, education, monthly expenditure, smoking, alcohol use, physical activity, BMI, health status, self-rated health | Impute from CHD death | N | Y |
| Capistrant et al, 2013 (17) | Cox proportional hazards regression | HR | Race, age, sex, SES, BMI, smoking status, physical activity, chronic conditions (high blood pressure, diabetes, cancer, lung disease, arthritis), heart medication use, psychiatric medication usage | Y | N | N |
| Gustad et al, 2014 (18) | Cox proportional hazards regression | HR | Cohabitation, smoking, physical activity, cancer, asthma, diabetes mellitus, endocrine disorders, musculoskeletal disorders, autoimmune disorders, epilepsy, blood pressure, BMI, serum cholesterol level | Y | N | N |
| Brunner et al, 2014 (19) | Cox proportional hazards regression | HR | Age, sex, ethnicity | Y | N | N |

Abbreviations: BMI, body mass index; CHD, coronary heart disease; COPD, chronic obstructive pulmonary disease; HR, hazard ratio; MI, myocardial infarction; N, no; OR, odds ratio; SES, socioeconomic status; Y, yes.

^a^ Combined MI and CHD death outcomes

**References**

1. Pratt LA, Ford DE, Crum RM, Armenian HK, Gallo JJ, Eaton WW. Depression, psychotropic medication, and risk of myocardial infarction: prospective data from the Baltimore ECA follow-up. Circulation. 1996 Dec 15;94(12):3123-9.

2. Penninx BW, Guralnik JM, Mendes de Leon CF, Pahor M, Visser M, Corti MC, et al. Cardiovascular events and mortality in newly and chronically depressed persons > 70 years of age. Am J Cardiol. 1998 Apr 15;81(8):988-94.

3. Ford DE, Mead LA, Chang PP, Cooper-Patrick L, Wang NY, Klag MJ. Depression is a risk factor for coronary artery disease in men: the precursors study. Arch Intern Med. 1998 Jul 13;158(13):1422-6.

4. Sesso HD, Kawachi I, Vokonas PS, Sparrow D. Depression and the risk of coronary heart disease in the Normative Aging Study. Am J Cardiol. 1998 Oct 1;82(7):851-6.

5. Whooley MA, Browner WS; Study of Osteoporotic Fractures Research Group. Association between depressive symptoms and mortality in older women. Arch Intern Med. 1998 Oct 26;158(19):2129-35.

6. Mendes de Leon CF, Krumholz HM, Seeman TS, Vaccarino V, Williams CS, Kasl SV, et al; Established Populations for the Epidemiologic Studies of the Elderly. Depression and risk of coronary heart disease in elderly men and women: New Haven EPESE, 1982-1991. Arch Intern Med. 1998 Nov 23;158(21):2341-8.

7. Penninx BW, Beekman AT, Honig A, Deeg DJ, Schoevers RA, van Eijk JT, et al. Depression and cardiac mortality: results from a community-based longitudinal study. Arch Gen Psychiatry. 2001 Mar;58(3):221-7.

8. Egede LE, Nietert PJ, Zheng D. Depression and all-cause and coronary heart disease mortality among adults with and without diabetes. Diabetes Care. 2005 Jun;28(6):1339-45.

9. Wulsin LR, Evans JC, Vasan RS, Murabito JM, Kelly-Hayes M, Benjamin EJ. Depressive symptoms, coronary heart disease, and overall mortality in the Framingham Heart Study. Psychosom Med. 2005 Sep-Oct;67(5):697-702.

10. Ladwig KH, Marten-Mittag B, Lowel H, Doring A, Wichmann HE. Synergistic effects of depressed mood and obesity on long-term cardiovascular risks in 1510 obese men and women: results from the MONICA-KORA Augsburg Cohort Study 1984-1998. Int J Obes (Lond). 2006 Sep;30(9):1408-14. Epub 2006 Mar 21.

11. Ahto M, Isoaho R, Puolijoki H, Vahlberg T, Kivela SL. Stronger symptoms of depression predict high coronary heart disease mortality in older men and women. Int J Geriatr Psychiatry. 2007 Aug;22(8):757-63.

12. Surtees PG, Wainwright NW, Luben RN, Wareham NJ, Bingham SA, Khaw KT. Depression and ischemic heart disease mortality: evidence from the EPIC-Norfolk United Kingdom prospective cohort study. Am J Psychiatry. 2008 Apr;165(4):515-23. Epub 2008 Feb 1.

13. Whang W, Kubzansky LD, Kawachi I, Rexrode KM, Kroenke CH, Glynn RJ, et al. Depression and risk of sudden cardiac death and coronary heart disease in women: results from the Nurses’ Health Study. J Am Coll Cardiol. 2009 Mar 17;53(11):950-8.

14. Janszky I, Ahnve S, Lundberg I, Hemmingsson T. Early-onset depression, anxiety, and risk of subsequent coronary heart disease: 37-year follow-up of 49,321 young Swedish men. J Am Coll Cardiol. 2010 Jun 29;56(1):31-7.

15. Brown JM, Stewart JC, Stump TE, Callahan CM. Risk of coronary heart disease events over 15 years among older adults with depressive symptoms. Am J Geriatr Psychiatry. 2011 Aug;19(8):721-9.

16. Sun WJ, Xu L, Chan WM, Lam TH, Schooling CM. Are depressive symptoms associated with cardiovascular mortality among older Chinese: a cohort study of 64,000 people in Hong Kong? Am J Geriatr Psychiatry. 2013 Nov;21(11):1107-15. Epub 2013 Feb 6.

17. Capistrant BD, Gilsanz P, Moon JR, Kosheleva A, Patton KK, Glymour MM. Does the association between depressive symptoms and cardiovascular mortality risk vary by race? Evidence from the Health and Retirement Study. Ethn Dis. 2013 Spring;23(2):155-60.

18. Gustad LT, Laugsand LE, Janszky I, Dalen H, Bjerkeset O. Symptoms of anxiety and depression and risk of acute myocardial infarction: the HUNT 2 study. Eur Heart J. 2014 Jun 1;35(21):1394-403. Epub 2013 Sep 20.

19. Brunner EJ, Shipley MJ, Britton AR, Stansfeld SA, Heuschmann PU, Rudd AG, et al. Depressive disorder, coronary heart disease, and stroke: dose-response and reverse causation effects in the Whitehall II cohort study. Eur J Prev Cardiol. 2014 Mar;21(3):340-6. Epub 2014 Feb 3.


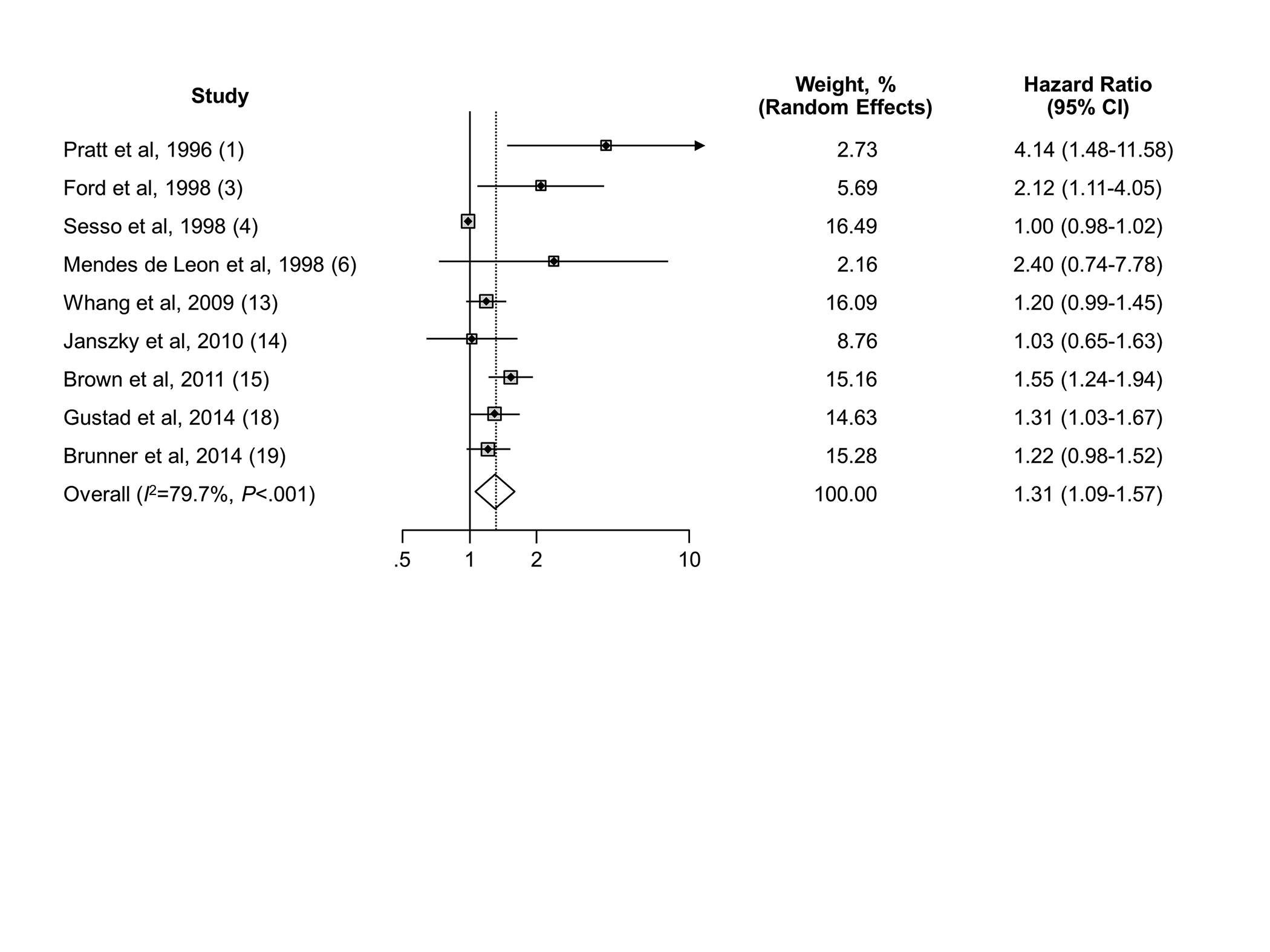


**eFigure 1.** Effect of Depression on the Risk of Myocardial Infarction. Hazard ratios (95% CIs) are shown; sizes of data markers are proportional to the weight of every study in the forest plot.


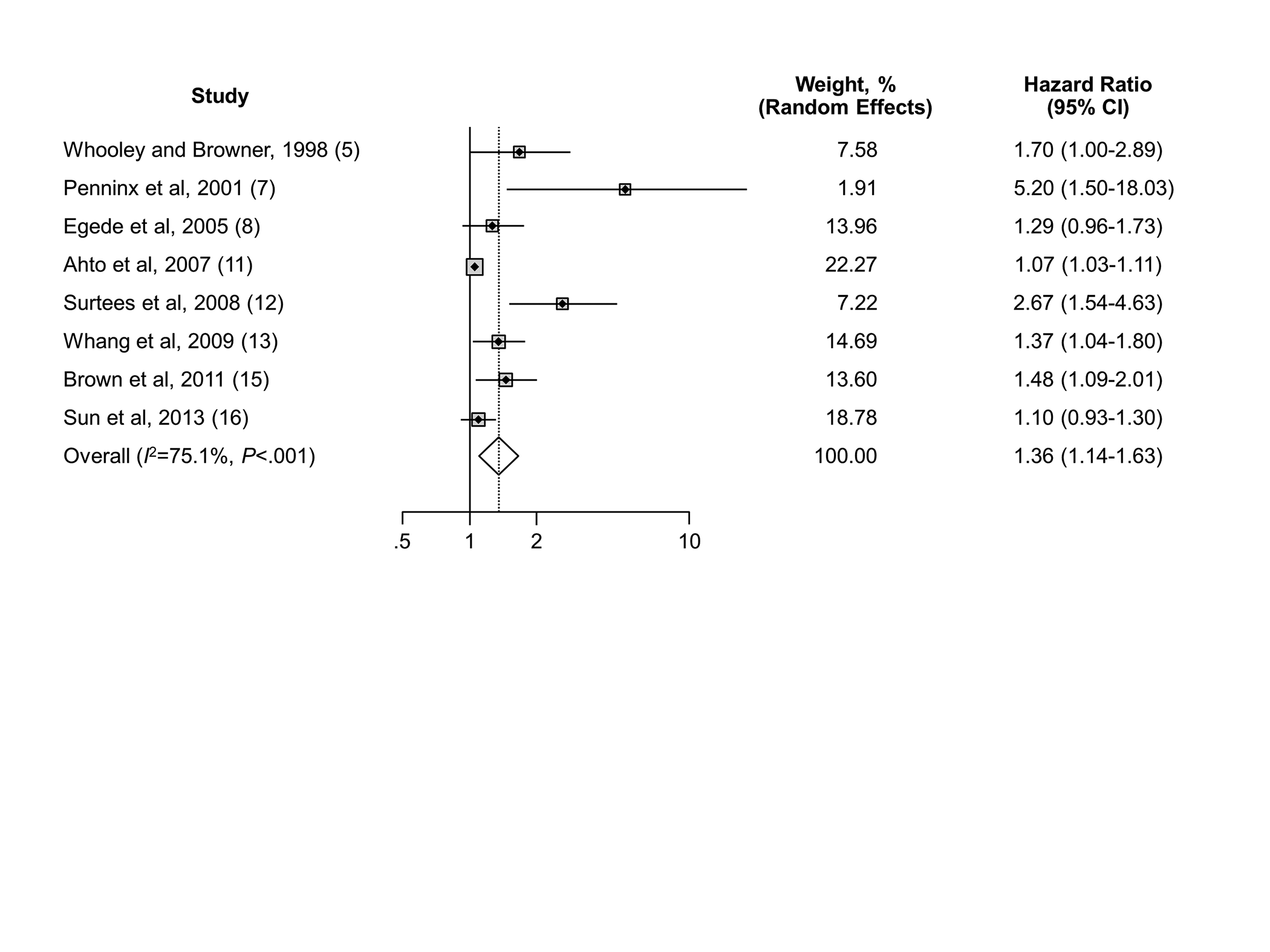


**eFigure 2.** Effect of Depression on the Risk of Coronary Death. Hazard ratios (95% CIs) are shown; sizes of data markers are proportional to the weight of every study in the forest plot.


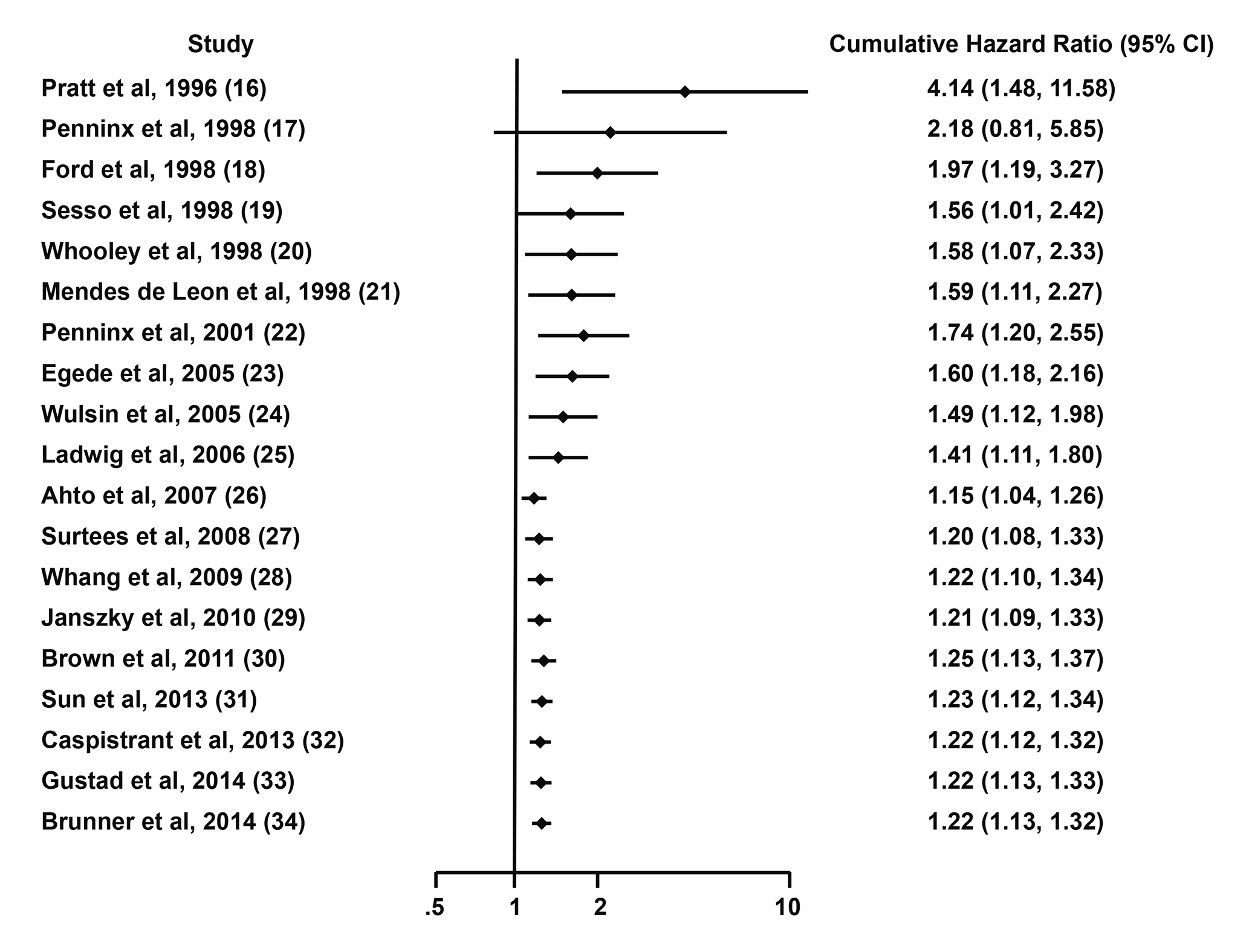


**eFigure 3**. Cumulative meta-analysis
